# Improved Bioavailability of Poorly Soluble Drugs through Gastrointestinal Muco-Adhesion of Lipid Nanoparticles

**DOI:** 10.3390/pharmaceutics13111817

**Published:** 2021-10-31

**Authors:** Sui Ling Janet Tan, Nashiru Billa

**Affiliations:** 1 Institut Kanser Negara, Putrajaya 62250, Malaysia; jtansuiling@hotmail.com; 2 College of Pharmacy, QU Health, Qatar University, Doha 2713, Qatar

**Keywords:** nanoparticle, nanostructured lipid carrier, solid lipid nanoparticle, muco-adhesion, polymer, gastrointestinal, bioavailability

## Abstract

Gastrointestinal absorption remains indispensable in the systemic delivery of most drugs, even though it presents several challenges that, paradoxically, may also provide opportunities that can be exploited to achieve maximal bioavailability. Drug delivery systems made from nanoparticle carriers and especially, lipid carriers, have the potential to traverse gastrointestinal barriers and deploy in the lymphatic pathway, which aptly, is free from first pass via the liver. Several poorly soluble drugs have presented improved systemic bioavailability when couriered in lipid nanoparticle carriers. In this review, we propose an additional frontier to enhancing the bioavailability of poorly soluble drugs when encapsulated in lipid nano-carriers by imparting muco-adhesion to the particles through application of appropriate polymeric coating to the lipid carrier. The combined effect of gastrointestinal muco-adhesion followed by lymphatic absorption is a promising approach to improving systemic bioavailability of poorly soluble drugs following oral administration. Evidence to the potential of this approach is backed-up by recent studies within the review.

## 1. Introduction

**Figure 1:**
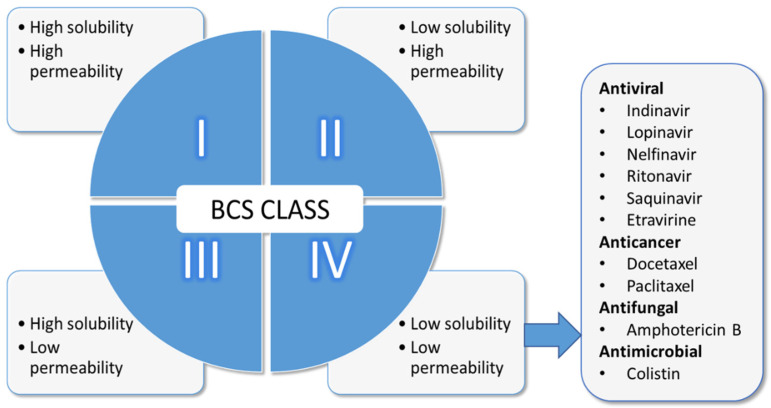
BCS classification system with examples of BCS Class IV drugs.

The oral route of administration remains most popular among patients because it is the most natural way to get medicines into the body. More than 60% of active pharmaceutical ingredients (API) are almost exclusively administered orally, and this represents a strong global share in the order of 90% of all dosage forms [1]. The popularity of the oral route of administration also correlates with patient compliance [2], yet, lower blood levels are observed compared to parenteral routes. This is a consequence of the epithelial barriers APIs must traverse before becoming systemically available as well as physicochemical properties of the API and physical factors related to the dosage form [3,4]. Furthermore, perturbation in systemic blood concentration occurs following first pass via the liver. The effect of food on gastrointestinal physiology, including altered gastric emptying times of dosage forms and physical interaction between the API and the food play a role in how much is presented at the target site. Apart from the effect of food on gastric emptying of dosage forms, food can alter the pharmacokinetics of APIs indirectly by interacting with gut-wall metabolizing enzymes. The best-known examples is altered pharmacokinetics of certain drugs when co-administered with grapefruit juice. This interaction is the result of inhibition of CYP3A4 metabolism as well as inhibition of the efflux transporters resident on the gut wall [5]. The consequence is an increase or decrease in bioavailability of drugs depending on whether the drug is a substrate to these enzymes. Food containing xenobiotics can also interfere with gut-wall metabolizing enzymes. Noteworthy are indole-containing vegetables such as cabbage and broccoli. Nonspecific alteration in bioavailability of APIs by certain foods may arise from physical interaction, for example dietary calcium ions (Ca^2+^) interacting with tetracycline or iron-containing formulations (F^2+^ or F^3+^) with tannic acid [6], both of which result in reduced bioavailability. High calorie diet can interfere with drugs absorption in a variety of ways. Poorly soluble drugs such as griseofulvin exhibits improved absorption when administered with high calorie diet, due to improved solubility in lipid milieu. On the other hand, fat receptors are believed to delay gastric emptying and hence, may impeded the rate of absorption of some drugs [6]. Besides, the Biopharmaceutics Classification System (BCS) developed by Amidon’s team [7] informs that the solubility of APIs in the GI tract and permeability through the GI membrane are both the crucial parameters that control the rate and extent of absorption (Figure 1).

Low solubility/high permeability and low solubility/permeability drugs (Class II and IV) are of great interest to scientists because they make up 70–90% of newly discovered APIs and about 40% of currently marketed dosage forms [4,8,9]. Research on orally administered APIs has traditionally focused mainly on Class IV, APIs, for example amphotericin B, furosemide, acetazolamide, saquinavir and ritonavir [4,10,11], due to the challenges that these drugs present following administration and subsequent traversing across the GI epithelia, which aptly, results in erratic absorption and low bioavailability [4,12].

The mucous layer within the gastrointestinal tract serves as a protective barrier to the underlying epithelia. One of the key component of the mucous layer is mucin and its effect on API bioavailability is often overlooked [13]. Mucin is either secreted or cell-bound [13] and the high content of sialic acid gives it a net negative charge. Secreted mucins are linked via disulfide (S=S) bonds to form macromolecules that entangle and give rise to the typical viscoelastic gel with shear-thinning properties [14]. They are primarily responsible for the gel-like appearance but the viscoelasticity is controlled by lipid, water and ion content [15]. The protective function of mucin over the underlying epithelia means that the turnover rate of adherent mucin must be balanced by production rate. In human, this balance maintains a thickness of approximately 200 µm in the small intestine, although this is highly variable [14]. The meshwork formed from the macromolecules creates random pores, with size in the order of 300 nm, which serves as a conduit for the delivery of submicron particles. Nanoparticles can serve as drug delivery carriers for gastrointestinal absorption of poorly soluble APIs [16,17,18]. In this regard, it is desirable that the nanoparticulate delivery systems be engineered to interact with the mucous layer but not to be withheld within the meshwork. Nanoparticles with very strong positive surface charge will strongly interact with mucin and be held up within, thus preventing traversing. On the other hand, nanoparticles possessing slightly positive or close to a net neutral charge will offer the required level of electrostatic interaction with mucin to prompt muco-adhesion but not muco-retention [13]. Favorable muco-adhesive interactions with mucin will promote passive conduit toward underlying epithelia.

The Payer’s patches within the gastrointestinal epithelia can phagocytose whole particles that deploy into the lymphatic system [19]. Thus, nanoparticles with adequate surface charge can be used to cargo poorly soluble APIs across the epithelia for improved systemic bioavailability. In this regard, lipid-based carriers such as solid lipid nanoparticles (SLN) or nanostructured lipid carriers (NLC) have a prime advantage for being lipidic and hence following a lymphatic trajectory whilst avoiding the first pass via the liver [20,21].

In this review, we present an observation from the literature that points to the proposition of utilizing a combined approach of nano-formulation through lipid carrier systems and muco-adhesion as a rational approach to improving bioavailability of APIs destined for gastrointestinal absorption.

## 2. Lipid-Based Nanoparticulate Drug Delivery

With the advent of nanotechnology, it is now possible to formulate APIs into suitable delivery systems, which possess requisite physicochemical properties for deployment via the gastrointestinal route (GI) in a safe and/or targeted manner.

Nanotechnology may be applied to address the principal constraints akin to poorly soluble/absorbable APIs, through improvement in (i) API solubilization (ii) muco-adhesion behavior of delivery system and (iii) cellular uptake of particulate delivery systems [22,23,24,25].

Lipid-based nanoparticulate delivery systems have gained increased attention by researchers in recent years because they are biocompatible, mostly biodegradable, and have wide biomedical applications [4,26,27]. Furthermore, they have proven commercial, pharmaceutical and therapeutic benefits for compounds such as cyclosporine A, lipid soluble vitamins and protease inhibitors [4,26,28]. They have the potential for use as drug delivery systems in various routes of administrations such as pulmonary [29,30], dermal [31,32,33], ocular [34,35,36], parental [37,38] and oral [25,27,39].

As per the remit of the present review, our focus is on the delivery of API via lipid-based nanoparticles via the GI tract. The nano-sized dimension coupled with the lipidic nature of the particles offers this class of nanoparticles superiority over other nanoparticulate delivery formulations. For example, lipid nanoparticles have better tolerability in vivo as they are biocompatible, biodegradable and capable of mimicking the natural digestive process of the dietary fat [11,27]. Additionally, the use of organic solvents during formulation can potentially be avoided, in contrast to polymer-based nanoparticles and thus, show safer toxicology profiles [26,30,40,41]. Besides, the absorption of poorly soluble APIs is enhanced by virtue of stimulation of biliary and pancreatic secretions by the particles [42,43], as evidenced by improved bioavailability of lipophilic vitamins (vitamin A, D, E and K), testosterone and halofantrine co-administered with fat-rich diet [22,30,44]. The simultaneous absorption of the drug along with the lipids is also known as the “Trojan Horse effect” [26,45].

Lipids offer protection to susceptible APIs that degrade via chemical, electromagnetic, oxidation and enzymatic reactions [26,30,40,41]. Moreover, lipid-based nanoparticles can alter the pharmacokinetic profiles of APIs through the manifestation of slow release behaviour from the delivery system [46]. Therefore, abrupt exposure to high drug concentration in vulnerable organs is minimised. In this context, the pharmacokinetic profile of API is now governed by the particle size, charge, type and concentration of the lipids rather than the intrinsic physicochemical properties of the API [47,48].

Due to their lipidic nature, this class of nanoparticles permit absorption of APIs through the lymphatic pathway [26,30,40,41], avoiding liver first pass and thus improving their systemic bioavailability [4,46,49].

Typical lipid-based nanoparticles are formulated as solid lipid nanoparticles (SLNs) and nanostructured lipid carriers (NLCs) [26,45,50,51]. Generally, they comprise of various types of physiological lipids with surfactants as stabilisers (Table 1) [30,51]. The following sections reviews the salient differences between SLNs and NLCs.

### 2.1. Solid Lipid Nanoparticles (SLNs)

SLNs are considered as the first-generation lipid-based nanoparticles. They are colloidal suspensions with particle size range in the order of 40–1000 nm and solid at both room and body temperatures and typically comprise of lipids as the matrix system (Table 1) [52,53].

SLNs possess all the advantages of lipid-based nanoparticles. For example, idarubicin-loaded SLNs showed a 21-fold increase in systemic bioavailability following oral administration, reflected through the area under the curve (AUC) and a 30-fold extended elimination half-life as compared to idarubicin solution [76]. In another study, cyclosporine A-loaded SLNs also showed high systemic bioavailability with no nephrotoxicity observed due to the slow release behavior from the SLNs compared to its marketed microemulsion formulation, Sandimmun^®^ Optoral/Neoral [26,77].

Despite these advantages, SLNs suffer from low API loading capacity with significant drug expulsion during storage, due to the polymorphic transformation of the lipids [22,30,53]. The poor loading capacity of SLNs is due to the densely packed lipid crystal lattice with few imperfections and thus, offering only little room for accommodation of APIs [30,31,53]. Hence, in order to address these SLN-related constraints, a second generation of lipid nanoparticles, nanostructured lipid carriers (NLCs) has emerged [4,22,26].

### 2.2. Nanostructured Lipid Carriers (NLCs)

NLCs formulations were developed in 2000 and within five years, two NLCs products were approved to be marketed (Nanorepair Q10 cream and Nanorepair Q10 serum, Dr. Rimpler, Wedemark, Germany) [78,79]. NLC represent nanocarriers with the shortest time between invention and marketing and considered very promising by the scientific community.

In contrast to the SLNs, NLCs formulations comprise of a blend of solid lipid with liquid oil (Table 1). Despite the presence of liquid oil, the NLCs retains the solid matrix at room and body temperature [40,80]. The liquid oil disrupts the crystallinity of the solid lipid matrix and thus, creates imperfections that allows incorporation high loads of API [12,22,81]. So, a five-fold increase in encapsulation efficiency (EE) of retinoids was observed upon inclusion of small amount of liquid oil into glyceryl behenate SLN matrix [82]. Similarly, an increase in EE was observed for AmpB encapsulated as SLNs from 52.77 to 75.33% when encapsulated as NLCs [83].

NLCs can be categorised as imperfect, amorphous or multiple NLCs according to the structure of the lipid matrix [51,84]. In imperfect NLCs there is distortion of the perfect crystal lattice due to the presence of a small amount liquid oils whilst amorphous NLCs are solid but non-crystalline state of lipids [51,84]. The amorphous NLCs are formed through the inclusion of liquid lipids such as hydroxyl octacosanyl, hydroxyl stearate, isopropyl myristate that prevent lipid crystallisation [48,51]. The multiple type NLCs contain numerous nano-sized liquid oil domains within the solid lipid matrix, so that the release of the API is prolonged because of the tortuous paths required to be traversed before release [51,84]. The composition and structure of NLCs allows high drug payloading, increased stability of the formulation upon storage [84].

Several highly lipophilic drugs have been encapsulated within NLCs for systemic bioavailability enhancement, most belonging to the BCS Class II, such as vinpocetine [58], lovastatin [80,85], simvastatin [86,87], atorvastatin [88], miconazole [89], quercetin [39,61], resveratrol [90], testosterone [91], tamoxifen [92], fenofibrate [93] and tacrolimus [94]. BCS Class IV encapsulated in NLCs include etoposide [95], saquinavir [96], oleate-docetaxel [97] and artemether-lumefantrine [88].

## 3. GI Uptake of Lipid-Based Nanoparticle Formulations

Sufficient GI uptake of the lipid nanoparticle along with its API cargo is the first and crucial step toward the systemic delivery of the API. However, it is important to recognize other potential barriers within the GI tract as well. For example, the mucosal layer as mentioned earlier, which serves as a protective layer to the underlying epithelia, presents a frontier that particulate delivery system must traverse. Fortunately, lipid nanoparticles have some tendency to adhere onto the mucosal layer and therefore able to oppose dislodging imposed by propulsive forces from GI motility [22,45]. Such shear forces will normally detach particulate delivery systems from the mucosa layer, but the smaller the particle size, the lower the translated shear stress from GI motility. Hence smaller sized particles are more likely to remain muco-adhered to the epithelia longer [98].

In a cell culture study on the permeability of NLCs across Caco-2 and HT-29 MTX co-culture, significant variation was observed between type of cell, where lower NLCs uptake occurred in cells without mucus (Caco-2) compared to HT-29 MTX, which expresses the mucosal layer [12].

Furthermore, charged particles may adhere electrostatically or be repelled from the mucosal layer depending on the resident surface charge on the particles, noting that, mucin is negatively charged due to sialic acid groups [99,100]. Although positively charged particles have strong muco-adhesion to mucin, they can potentially be entrapped within the layer and thus unable to effectively traverse the underlying epithelial cells [12,99]. This entrapment could be beneficial eventually, because prolonged GI transit time, improves the chances for lateral incursions toward uptake of particles the epithelia [101,102].

Upon oral ingestion, part of the lipid nanoparticles may become degraded or digested by gastric lipase aided by shear forces (e.g., propulsion, grinding and retropulsion) in the stomach, which forms a crude emulsion [4,103]. In the small intestine, the lipids may become degraded by enzymes (e.g., lipase-colipase complex) forming surface active mono-, diglycerides and fatty acids [4,22,26,84]. The presence of exogenous lipids stimulates the secretion of biliary lipids from the gall bladder, including bile salts, phospholipids and cholesterol, which react with and disperse the surface active mono-, diglycerides and fatty acids into fine oil droplets or micelles [4,26,45,103]. The micelles are incorporated into a series of colloidal structures, including mixed-micelles, unilamellar and multi-lamellar vesicles [4,22,26,45]. Prior to the formation of these colloidal structures, the API initially encapsulated within the NLC may be taken up or solubilised into the micelles [26,27,84]. Through that, the drugs are simultaneously taken up by the epithelial cells during the absorption process of the mixed-micelles [22,26,45].

Furthermore, the colloidal structures or some intact lipid nanoparticles can either be taken up through membranous epithelial cells (M-cells) or gut enterocytes [84,104]. M-cells are specialised epithelial cells, consisting of only 10% of cells on the dome of Peyer’s patches (PP) [22,51,105]. PP are gut-associated lymphoid tissues that comprise of aggregated or isolated lymphoid follicles and they provide entry points to the lymphatic system [76,106]. They possess high transcytotic capacity and able to transport a broad range of materials including macromolecules and microbes thus, can be exploited for the delivery of drugs, vaccines and bioactive materials [101,107,108].

The uptake of lipid nanoparticles into gut enterocytes is mainly through the transcellular active transport of endocytosis [76,106,109]. Endocytosis is internalisation of macromolecules into transport vesicles, derived from the plasma membrane [110,111]. It can be further categorised as phagocytosis or pinocytosis [23,109,112]. Phagocytosis involves M-cells and immune cells such as macrophages, dendritic cells, monocytes and nucleophiles [106,109,111]. Unlike phagocytosis, pinocytosis is not restricted to specialised cells but could occur in any type of cells [23,112].

Pinocytosis is more complex and may include clathrin-mediated endocytosis (CME), caveolae-mediated endocytosis (CvME), macropinocytosis, clathrin or caveolae-independent endocytosis. CME occurs in clathrin-enriched membrane and includes receptor-ligand interactions [109,112]. CME leads to the formation of early endosome, late endosome and finally lysosome. The lysosome has an acidic (pH 4.5–5.5) and is an enzyme-rich environment thus, may promote degradation of labile therapeutic agent or nanocarriers [108,109,112].

CvME involves cholesterol-rich or sphingolipids membranes that are lined with caveolin, a dimeric protein with a flask-shape membrane invaginations characteristic [112,113]. Caveolae vesicles may protect the nanoparticles from lysosomal degradation, thus, it is a preferred pathway for delivery of enzymes and proteins in contrast to the CME pathway [108]. However, CvME occurs at a slower rate than CME pathway due to its highly regulated process that involves complex signaling mechanisms [110,112].

Similar to phagocytosis, macropinocytosis involves protrusion and fusion of the membrane, encapsulating the particles and forms vesicles of about 1 µm. However, the process of macropinocytosis is non-selective and highly dependent on the difference in the solute concentration [108,111].

Lipid nanoparticles can also traverse the paracellular route after transient disruption of tight junctions between adjacent epithelial cells by permeation enhancers [76,84,105]. However this mode of absorption is highly variable and dependent on the permeation enhancer but typically up to 2000 kDa opening is possible as evidenced by permeation of Flourescein isothiocynate (FICT) [114]. The tight junctions are modulated by proteins such as junctional adhesion molecules (JAM), occludins and claudins that regulate the passage of particles. Studies have shown that some polymers like chitosan [99,115] and thiolated polymers [116] are capable of reversibly disrupting the tight junctions, and thus, could be further exploited for the transport of macromolecules or nanoparticles. However, transcellular route remains the predominant passage route as the fraction of epithelial cells are greater than the percentage of tight junctions [109,117].

After the formed micelles are taken up by the enterocytes cells, they become converted into chylomicrons upon re-esterification via monoacyl glycerol or phosphatidic acid pathway and subsequently, stabilised by phospholipids [4,76,118]. Finally, the formed chylomicrons are transported via the intestinal lymphatic pathway [4,119].

The lymphatic system is an additional pathway for the absorption of lipid nanoparticles or other lipophilic compounds (e.g., long-chain fatty acids, cholesterol esters and fat-soluble vitamins), in contrast to most orally administered drugs, which are transported mostly via the portal blood vein before reaching the systemic circulation [51,120]. The lymphatic system consists of lymph, capable of maintaining the homeostasis through the regulation of extracellular fluid and helps in body defense system by transporting immune cells to injury sites [76,121].

Lipid nanoparticles that are absorbed by the intestinal lymphatics are transported through the mesenteric lymph duct that enter into the thoracic duct and empty in the systemic circulation via left jugular and subclavian veins [102,122]. The lymphatic system is a formidable absorption pathway for drug delivery because it (i) bypasses the first pass metabolism that increases drug bioavailability, (ii) has prolonged drug delivery due to longer duration of drug transport and (iii) offers the possibility of targeting drugs to lymph, potential application in lymphatic cancers and relevant infections such as leishmaniasis, malaria and AIDS [26,51,76,102,105].

Lymphatic absorption of lipid nanoparticles has been extensively explored as a viable means for delivering poorly soluble APIs. A commercial formulation of testosterone (Andriol^®^) dissolved in oleic acid has been exploited for lymphatic absorption which accounted for 91.5% of the total bioavailability [123]. In another study, methotrexate-loaded SLNs showed a 10-fold increase in bioavailability attributable to the lymphatic system [60]. Furthermore, Khan et al. (2013) reported that vincopecetine-loaded NLCs observed a two-fold increase in the C_max_ compared to pure vincopecetine solution. The lymphatic pathway was hypothesised to be the main transportation route for the NLCs as opposed to the solution, which suffered significant first pass [76].

## 4. Importance of GI Muco-Adhesion

Conventional formulations often face challenges due to inability to withstand the strong involuntary muscular contractions and the washing effects due to GI luminal content [124]. This limitation results in the loss of crucial amounts of administered API, and thus, necessitates multiple dosage administration in order to achieve blood therapeutic levels required to elicit pharmacological responses [34,125]. Such API washing can potentially result in the failure of the therapy and result in an overall increase in treatment costs [124].

**Figure 2:**
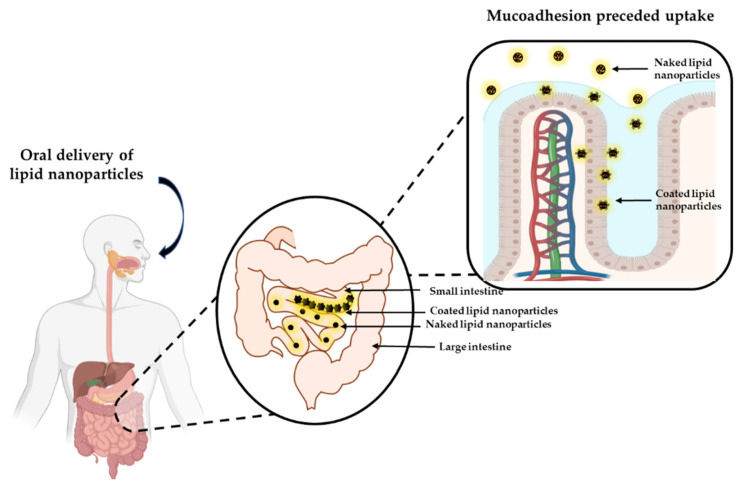
Schematic diagram of muco-adhesion behavior of lipid nanoparticles. Created with Biorender.com and ChemDraw Professional.

Muco-adhesive GI delivery systems can adhere to the mucous layer of the GI epithelia and hence prolong the residence time of the formulation at the absorption site (Figure 2) [100,126]. This intimate contact with the epithelium confers a higher permeation propensity which, subsequently increases the systemic bioavailability of the API [124,127]. Nanoparticulate delivery systems are inherently muco-adhesive [32,33,34,35], however, this property may be enhanced further by coating the particles with appropriate polymers. Such coated particles also protect labile drugs from the GI milieu so that unaltered form of the API traverses across the epithelia [124,128].

GI muco-adhesion by nanoparticulate dosage may occur via a variety of mechanisms including: (i) electronic, (ii) wetting, (iii) adsorption, (iv) diffusion and (v) mechanical. The electronic phenomenon is based on the establishment of attractive forces through the transfer of electrons (e.g., between cationic polymer and negatively charged mucin), forming an electrical double layer [100,127,129]. In the wetting mechanism, a measure of the spreadability of the dosage form on the mucous layer, and hence the magnitude of its contact angle (immersional contact angle) to the mucus layer is used to assess muco-adhesivity. Lower contact angles indicate better propensity to muco-adhesion [100,127,129]. The adsorption mechanism is based on semi-permanent interactions such as hydrogen bonds, van der Waals and hydrophobic interactions as the driving forces for muco-adhesion and they require less energy to detach. Based on diffusion mechanism, adhesive forces are established through a time-dependent diffusion of the polymer chains into the glycoprotein chain network of the mucosal layer [127,130]. The mechanical theory proposes that adhesion occurs because of the diffusion of the polymer into the irregularities of the mucosal surface, which increases the interfacial area for interactions [100,126,131].

Taking advantage of the lymphatic pathway, lipid-based delivery systems, and especially submicron carriers, provide an avenue for the deployment of payloads via the lymphatic route into the systemic circulation. Whilst this is true in in vitro and animal models, GI delivery of lipid-based systems still fall short in the clinical settings and therefore warrants attention [132]. Oral delivery of AmpB has been widely investigated and includes polymeric nanoparticle carbon nanotubes, nanosuspensions, polymer lipid hybrid nanoparticles, solid lipid nanoparticles (SLN), cubosomes, emulsions and cochleates [20,67,133,134,135]. In the work by Amekyeh et al. (2015), the extent of absorption, rather than the rate from the GI tract, was the key contributor to improved bioavailability of amphotericin B [20]. This is indicative of uptake via the lymphatic route.

Within the context of the present review, attendant muco-adhesion imparted by the delivery system appear to be a rational approach for achieving both localized therapeutic action and systemic delivery through uptake by enterocytes. SLN uptake via the gastrointestinal tract can be enhanced if the transit of the particles can be extended. SLN and NLCs are inherently neutral or exhibit negative charge potential due to the lipids. However, it is possible to impart positive potential to SLN or NLC from positively charged surfactants, such as cetyltrimethylammonium bromide (CTAB). Although muco-adhesivity is achievable from such particles, toxicity concerns limit the use of positively charged surfactants [136], especially for internal applications. On the other hand, cationic lipids such as cationic lipid *N*, *N*-dioleyl-*N*, *N*-dimethylammonium chloride (DODAC) can be formulated as cationic SLN or NLC but toxicity remains a concern [137]. In addressing toxicity concerns, SLNs or NLC can be coated with muco-adhesive polymers that exhibit low or no toxicity to cells. Chitosan, a natural, non-toxic, biocompatible polycationic polysaccharide, derived from partial deacetylation of chitin is well studied and reported to possess muco-adhesive properties [132]. SLN have also been investigated for deployment in the small intestinal region for systemic absorption [73] or the colonic region of the gastrointestinal tract in the management of colon cancer, whereby doxorubicin and docetaxel were primed for local delivery [138]. In order to facilitate a controlled release of docetaxel SLN have been coated with glycolic acid [139], which is likely to impart some degree of muco-adhesion. In terms of muco-adhesion for therapeutic applications, folic acid-grafted on SLN containing irinotecan were encapsulated as microbeads of alginates and coated with Eudragit S100, which is a pH responsive enteric polymer [140,141,142]. It swells and dissolves at resident colonic pH values. This colon-targeting capability of the SLN was confirmed via a radiolabeled biodistribution study, where the microbeads showed a residence time of over 48 h in cancer tissue [142]. Thus, oral delivery of the SLN/microbeads enhanced anti-colon cancer efficacy. A high local GI concentration of SiRN was reported when the latter was delivered orally as lipoidoid nanoparticles through muco-adhesion [143]. The authors add that SiRN delivery to immune cells is possible by this approach. Furthermore, bees wax modified with phospholipids had shown muco-adhesion attributes and thus used to enhance mucosal delivery of antifungal drugs such as miconazole nitrate [144]. Lou et al. (2015) showed that the uptake of coumarin 6-loaded chitosan coated SLN by Caco2 cells was 44% in contrast to 10% from non-coated SLN [125]. Enhanced absorption was also observed from Caco2 monolayers of an insulin containing chitosan coated SLN. However, further improvement in insulin absorption was observed from HT29 cells, which are mucus producing. Oral administration of the chitosan coated SLN to diabetic rats resulted in a significant hypoglycemic effect in rats [145]. Amphotericin B containing chitosan-coated NLC demonstrated significant improvement in bioavailability after oral administration in rats compared to uncoated NLC or Amphotret^®^ [18]. In a related study, chitosan coated NLC displayed biocompatibility with enhanced antifungal properties compared to uncoated NLC [146].

## 5. Muco-Adhesion Polymers

Muco-adhesion polymers can be classified as non-ionic, anionic or cationic. Non-ionic polymers (e.g., polyethylene oxides) exert their muco-adhesion through a buildup of the hydrogen bonds and entanglement of the polymer chains, independent of the surrounding pH conditions. However, they were reported to be less adhesive as compared to anionic or cationic polymers [130,147]. Anionic polymers such as sodium carboxymethylcellulose (NaCMC) and poly (-acrylic acid) (PAA) with its derivatives, exert their muco-adhesion behaviour through hydrogen bonding via their carboxyl functional groups with the hydroxyl groups of the oligosaccharide side chains of the mucus protein [100,130]. The disadvantage of anionic polymers is that they tend to precipitate in the presence of multivalent cations like Mg^2+^ and Ca^2+^ and which reduces their muco-adhesion capacity [130].

Cationic polymers exhibit muco-adhesion behavior through ionic interactions between polymers and the anionic sialic acid groups of the mucus [68,124]. Among the cationic polymers, chitosan is undoubtedly the most extensively investigated in the literature [129,130], owing to its biocompatibility and biodegradability.

## 6. In-Vitro/Ex Vivo Muco-Adhesion Studies

The muco-adhesion properties of conventional dosage forms or materials have been preferentially measured using methods such as tensile and shear strength tests, which directly measures the time or force needed to detach the dosage form from a model membrane [127]. These methods may involve different detachment techniques but overall they are easy and fast to perform [100,148]. However, those tests impose limitations due to their bulky nature and as such difficult to ascertain whether the detachment has occurred at muco-adhesion interface or simply due to the loss of cohesion between polymers molecules or mucin components. Additionally, the surface properties of nanosystems (e.g., morphology, curvature and polymer disposition) as well as the closer proximity of the interactions of the nanosystem with the mucosal environment, justify the development of techniques used to evaluate muco-adhesion at nanoscale [100,127].

The following sections will focus on the different experimental approaches used to assess the muco-adhesion potential of nanoformulations, which can be classified as indirect or direct methods (Table 2). 

Indirect methods evaluate the balance between contributing and detrimental interactions between the nanoformulation and mucins whereas direct methods evaluate muco-adhesion in vivo or using biological tissue (ex vivo).

### 6.1. Indirect Methods

#### 6.1.1. Mucin Particle Method

Mucin particle method is an indirect method used to assess the muco-adhesion properties of the nanoformulation. It evaluates the degree of adsorption of nanoparticles to mucin particles through the measurement in the variation of size [149], zeta potential or electrophoretic mobility of the formed complexes [150,151].

Besides, the interaction between mucin in solution and nanoparticles could also be assessed through turbidity concept whereby the transmittance [152] or absorbance [146,153] of the dispersion after an adequate incubation time is measured. The interaction between nanoparticles and mucin will form macro aggregates, which scatters light more intensely and thus increases the turbidity of the sample [127,154]. Although mucin particle method is an easy and cheap method, additional studies are needed since higher concentrations of mucin than those observed in in vivo are often reported [127].

#### 6.1.2. Microgravimetric Method

Microgravimetric method evaluates the amount of material adsorbed onto the mucin using a quartz crystal microbalance [155]. The muco-adhesive behavior is evaluated through the changes in the resonance frequency calculated using the Sauerby equation [156]. It is a sensitive method and allows real-time data to be acquired, providing valuable kinetic profile of muco-adhesion process. However, there is a lack of published data to prove the real value of this method.

#### 6.1.3. Atomic Force Microscopy (AFM)

AFM measures the attractive and repulsive forces exerted at molecular level through the modification of the probe tip with polymer or mucin. Interaction with different samples (e.g., cell layers) will result in deflection the cantilever attached to the probe and thus, the interaction forces (both attractive and repulsive) can be measured [157]. Besides, AFM allows measurement to be carried out in milieu mimicking biological systems in real-time data. However, cost, complexity and reproducibility are the major disadvantages of this method [127].

**Table 2:** Methods used to evaluate the muco-adhesion properties of the formulations and comparison of their key features.

Techniques	Principle of the Technique	Mechanism	Real Time	Relevant	Feasibility	Cost	References
*Indirect*							
Mucin particle	Determined through variation in size, ζ and turbidity.	+	No	+	+++	+	[149,158]
Microgravimetric	Evaluation of material adsorbed using quartz crystal microbalance based on resonance frequency.	++	Yes	+	++	++	[125,155]
AFM	Probe tip modified with polymer/mucin, measure attractive/repulsive forces exerted at molecular level.	+++	Yes	+	+	+++	[157,159]
Optical techniques	Changes in properties of incident light on surface immobilised mucin on binding with nanoparticle (ellipsometry and surface plasmon resonance).	++	Yes	+	++	+++	[150,160]
Diffusion/particle tracking	Impediment to the unhindered diffusive movement of nanoparticles (multiple particle tracking).	+++	Yes	++	+++	++	[161]
*Direct*							
Cytoadhesion	Adhesion of fluorescent-labelled nanoparticles in cell culture using fluorescent microscopy.	++	Optional	++	++	++	[160]
Ex vivo methods	Retention of labelled nanoparticles in mucosal tissue/based on weight difference of nanoparticles.	+	Optional	++	++	++	[162]
In vivo administration/ ex vivo analysis	Administration of labelled nanoparticles to living animals, evaluation upon sacrifice.	+	No	+++	++	++	[163,164]
In vivo imaging	Natural trafficking of labelled-particles (e.g., barium sulphate, technetium-99 m).	+	Yes	+++	+	+++	[29,165]

#### 6.1.4. Optical Technique

In the literature, several optical techniques employ changes in properties of incident light on surface immobilised mucin when nanoparticles adsorbs to the mucin. For example, ellipsometry measures the muco-adhesion (weight of nanoparticles adsorbed per area) based on the refractive index from the mucin layer. This is shown through the study by Svensson et al. (2008), utilising chitosan-modified particles and cubosomes on mucin coated surfaces [150]. 

Surface plasmon resonance optical technique utilises a resonant mirror biosensor to evaluate changes in the refractive index based on evanescent waves. The association between the nanoparticles and mucin can be obtained through the mathematical model utilising the quantitative data produced [155].

#### 6.1.5. Diffusion/Particle Tracking 

Multiple particle tracking (MPT) has been extensively used to measure the interaction between unhindered diffusive movement of nanoparticles and mucus or mucus simulating fluid. Briefly, the fluorescent nanoparticles are incubated with mucus and placed under video microscopy to obtain high resolution 2D trajectories. The muco-adhesion properties can be evaluated based on quantitative parameters such as diffusivity/immobility and transport mode of tracked nanosystems [127,161].

### 6.2. Direct Methods

#### 6.2.1. Cyto-Adhesion

Generally, this type of study evaluates the adhesion of fluorescent labelled nanoparticles in epithelial cell monolayer using fluorescent microscopy. It is simple to execute but suffers from disadvantages such as lack of tissue architecture and functionality [160]. Hence, a more refined cell model was proposed, using a co-culture of intestinal epithelial cells (Caco-2) and mucus producing cells (HT-29). Study by Prego et al. (2006) demonstrated that chitosan nanocapsule showed a higher degree of association to co-culture of Caco-2/HT-29 compared to monoculture of Caco-2, revealing the muco-adhesive properties of chitosan [166].

#### 6.2.2. Ex Vivo Muco-Adhesion Method

**Figure 3:**
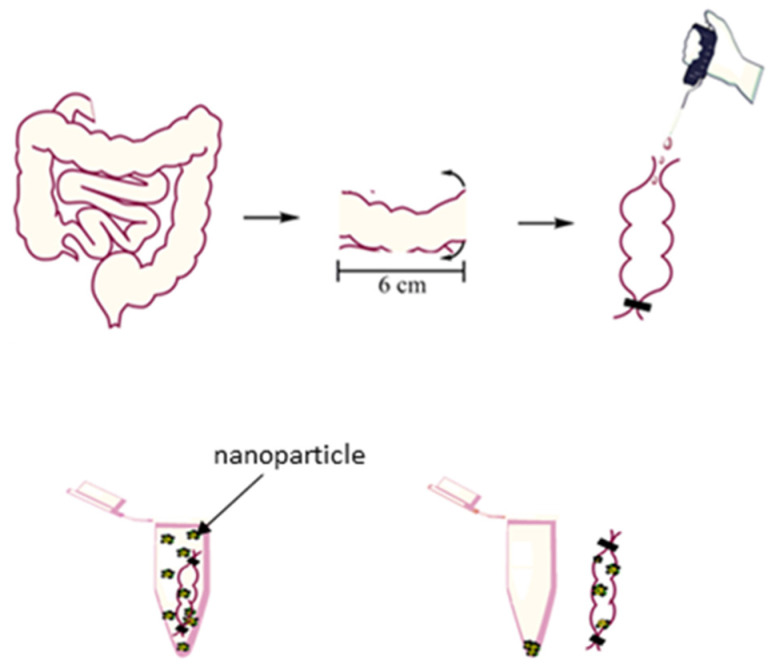
Ex vivo estimation of muco-adhesion propensity.

The ex vivo muco-adhesion method evaluates the degree of retention of radiolabeled or fluorescent-labelled nanoparticles in mucosal tissue explants [167]. Simpler method eliminates the need for additional radioisotopes or fluorescence in the nanosystem as such utilization requires a balance between the weight of the initially applied nanoparticles and remaining upon incubation with the mucosal explants [154,162]. The advantages of this method are that it utilizes natural mucus and is thus more physiologically relevant as well as less ethically ambiguous as it allows higher number of experiments to be performed [127]. With regard to ex vivo muco-adhesion of lipid nanoparticles, the particles can be suspended in an everted sac and the amount adsorbed estimated, Figure 3.

#### 6.2.3. In Vivo Administration/Ex Vivo Analysis

This method involves administration of fluorescent compounds (e.g., Cy 5.5 [168], sulforhodamine B [80] and coumarin-6 [99] or radioisotope-labelled (e.g., technetium-99 m) nanoparticles to the living animals following which the muco-adhesion is evaluated through harvesting relevant region of the GI tract in sacrificed animals over time (Table 2) [29]. The amount of the nanoparticles present at the region-of-interest is evaluated qualitatively using confocal microscopy [99] or quantitatively through fluorometric assay or gamma counter [165].

The major limitation to this method is the ethical constraints associated with the number of animals required for the experiment. Nevertheless, this technique allows good representation of the physiological washing, which is absent in the indirect and ex vivo methods as such could potentially influence the adhesion and retention of the nanoparticles [127].

## 7. Application and Future Perspectives of Muco-Adhesion Lipid Nanoparticle

Lipid nanoparticulate carriers provides a means for deploying APIs cargoes systemically, when delivered orally, by virtue of lymphatic uptake of the particles. In this regard, the bioavailability of several class III or IV drugs have been improved when formulated as orally administered lipid nanoformulations. Further improvement in systemic bioavailability can be achieved through delayed GI transit of the particles, when the latter is coated with muco-adhesion polymers. It is the view of the authors that more of the above classes of drugs that previously were delivered by alternative routes will now be prospective candidates for oral delivery via lipid nanoformulation cum GI muco-adhesion. Furthermore, lipid polymers that possess muco-adhesion properties should be investigated as possible carrier systems. Chemical conjugation of lipid carriers to muco-ahesive polymers will be a rational approach for use in nanoformulation. Muco-adhesion of nanoparticles as semisolid dosage form will find wide applications on mucosal surfaces for the treatment of localized diseases. In regard case, gradual release of the API from the resident formulation provides an effective therapeutic supply to concerned tissue. 

## 8. Conclusions

Lipid nanoformulation presents an alternative means to delivering poorly soluble APIs to the systemic circulation following oral administration and GI absorption, noting that insignificant bioavailability results from administration of such pure APIs. This review captures evidence supporting the emergence of an additional dimension in the utilization of lipid nanoformulation in for enhancing the systemic bioavailability of the API. This can be achieved through use of suitable polymers adequately adsorbed to the lipid nanoparticle in order to impart muco-adhesivity, thus extending the GI transit times. This allows lateral incursions across the epithelia ultimately feeding into the lymph. The combined approach works better in enhancing the bioavailability of the API than either used alone. One area where this approach will find wide application is the treatment of local conditions within the GI tract whereby lipid nanocarriers deploy through muco-adhesion and gradual release of the API.

## Figures and Tables

**Table 1:** Composition of SLNs and NLCs.

Class	Examples	References
**Solid lipids**	Cetylpalmitate	[54,55]
	Glyceral behenate (Compritol 888 ATO)	[56,57,58]
	Glyceryl monostearate	[55,58,59,60]
	Glyceryl tripalmitate	[61,62]
	Stearic acid	[63,64,65]
	Tristearin	[60,66]
	Beeswax	[18,20,67,68]
**Liquid oils**	Coconut oil	[18,68]
	Oleic acid	[63,69,70]
	Miglyol 812	[56,58,59]
	Castor oil	[70,71]
**Surfactants**	Poloxamer 188 (Pluronic^®^ F-68)	[57,58,59,72]
	Polysorbate 20 (Tween-20)	[73,74]
	Polysorbate 80 (Tween-80)	[62,75]
	Sodium cholate	[20,69]
	Sodium glycocholate	[59]
	Sodium taurocholate	[56,64]
	Sodium dodecyl sulfate	[63]
	Soybean lecithin	[20]
